# How do trial teams plan for retention during the design stage of the trial? A scoping review

**DOI:** 10.1186/s13063-023-07775-2

**Published:** 2023-12-04

**Authors:** Ellen Murphy, Katie Gillies, Frances Shiely

**Affiliations:** 1 Health Research Board - Trials Methodology Research Network (HRB-TMRN), Galway, Ireland; 2 Trials Research and Methodologies Unit (TRAMS), Health Research Board Clinical Research Facility University College Cork, Cork, Ireland; 3 Health Services Research Unit, University of Aberdeen, Aberdeen, UK; 4 School of Public Health, University College Cork, Cork, Ireland

**Keywords:** Scoping review, Randomised controlled trial, Protocol, Reporting, Retention, Methodology

## Abstract

**Background:**

Retention to trials is important to ensure the results of the trial are valid and reliable. The SPIRIT guidelines (18b) require “plans to promote participant retention and complete follow-up, including list of any outcome data to be collected for participants who discontinue or deviate from intervention protocols” be included in trial protocols. It is unknown how often protocols report this retention information. The purpose of our scoping review is to establish if, and how, trial teams report plans for retention during the design stage of the trial.

**Materials and methods:**

A scoping review with searches in key databases (PubMed, Scopus, EMBASE, CINAHL (EBSCO), and Web of Science from 2014 to 2019 inclusive) to identify randomised controlled trial protocols. We produced descriptive statistics on the characteristics of the trial protocols and also on those adhering to SPIRIT item 18b. A narrative synthesis of the retention strategies was also conducted.

**Results:**

Eight-hundred and twenty-four protocols met our inclusion criteria. RCTs (*n* = 722) and pilot and feasibility trial protocols (*n* = 102) reported using the SPIRIT guidelines during protocol development 35% and 34.3% of the time respectively. Of these protocols, only 9.5% and 11.4% respectively reported all aspects of SPIRIT item 18b “plans to promote participant retention and to complete follow-up, including list of any outcome data for participants who discontinue or deviate from intervention protocols”.

Of the RCT protocols, 36.8% included proactive “plans to promote participant retention” regardless of whether they reported using SPIRIT guidelines or not. Most protocols planned “combined strategies” (48.1%). Of these, the joint most commonly reported were “reminders and data collection location and method” and “reminders and monetary incentives”. The most popular individual retention strategy was “reminders” (14.7%) followed by “monetary incentives- conditional” (10.2%). Of the pilot and feasibility protocols, 40.2% included proactive “plans to promote participant retention” with the use of “combined strategies” being most frequent (46.3%). The use of “monetary incentives – conditional” (22%) was the most popular individual reported retention strategy.

**Conclusion:**

There is a lack of reporting of plans to promote participant retention in trial protocols. Proactive planning of retention strategies during the trial design stage is preferable to the reactive implementation of retention strategies. Prospective retention planning and clear communication in protocols may inform more suitable choice, costing and implementation of retention strategies and improve transparency in trial conduct.

**Supplementary Information:**

The online version contains supplementary material available at 10.1186/s13063-023-07775-2.

## Background

Retention of participants to trials is an ongoing challenge with little evidence to support what works and what does not work [1]. The most recent Cochrane systematic review of strategies to improve retention in trials found that there were no strategies that improved retention for which the quality of evidence was high. Despite this, many are used in trials frequently [1, 2] with some trials evaluating multiple retention strategies simultaneously [1]. We recently conducted a study to investigate how much the most routinely used trial retention strategies cost trial teams in the UK and Ireland. Even when calculated conservatively, the financial cost is staggering [3]. Even more staggering, it is estimated that roughly 50% of trials experience loss-to-follow-up of at least 11% with some experiencing loss-to-follow-up rates as high as 20% [1, 4]. Higher rates of loss-to-follow-up are shown to be associated with longer length of follow-up [5]. These statistics suggest that trial teams should consider retention strategies at the design stage, before the trial begins, particularly for trials at higher risk of loss-to-follow-up.

Poor retention causes bias to be introduced into the trial [5] and reduces the power of the trial which means the ability to detect significant findings and the confidence in the conclusions drawn from the trial are both affected [5–7]. Poor retention also results in incomplete data, it can delay the delivery of interventions and increase the costs associated with running trials [8]. This contributes to research waste [8–10]. Missing data and poor retention can be dealt with by statistical techniques in the analysis of the trial [5, 6], but no missing data technique is as good as retaining the participant and having complete data. Recruiting larger numbers of participants to counteract the expected dropout rate is also used to mitigate missing data, but this is more expensive and exposes more people to the risks associated with trial participation [11]. Rather than dealing with the problem after it occurs, trial teams could/should be looking to factor in plans to mitigate poor retention at design stages—a question identified as a priority for research by the trials community [12].

Trial protocols are an essential document for planning and conducting the trial. Protocols are reviewed and approved by ethics committees before the trial begins to ensure the trial team has fully accounted for any potential issues that may arise during the course of the trial [13]. Having a comprehensive clearly written protocol increases the transparency in trial conduct [13, 14] and allows for the replication of trial methods [14]. Protocols need to be published and be freely assessable for the readers of the corresponding results paper to fully appraise and interpret the results of the trial [15, 16]. Despite the importance of trial protocols, research shows that the content of protocols varies greatly [13, 14]. They often fail to report, in sufficient detail, some key trial design elements such as the primary outcome of the trial [17], statistical methods [18], and allocation concealment [19]. Deficiencies in protocol content may result in trial teams seeking ethical amendments, and may lead to poor trial conduct [13].

According to ICH GCP guidance, there is no requirement or recommendation that retention strategies be included in trial protocols but it does recommend that protocols should specify “the type and timing of the data to be collected for withdrawn subjects” ([20]:40). The new Clinical Trial Regulation [21] makes no comment on retention either. However, the U.S. Food and Drug Administration (FDA) recommend that preventing poor retention needs to be dealt with by improving trial design and trial conduct [22]. One document, developed to improve the completeness and reporting of content of trial protocols, SPIRIT (Standard Protocol Items: Recommendations for Interventional Trials) was developed in 2013. The SPIRIT statement is a 33-item checklist for minimum protocol content that aims to promote and improve the transparency and description of trial activities by encouraging trial teams to consider potential and important issues during the design stage of the trial [13]. One of these issues is retention, and as per the SPIRIT 2013 statement it is recommended that the following be included “plans to promote participant retention and complete follow-up including list of any outcome data to be collected for participants who discontinue or deviate from intervention protocols” ([14]:3). Existing evidence, from interviews with trial staff, as to whether trial teams actually prospectively plan retention strategies during the trial design stage is variable [23].

The purpose and primary aim of our scoping review is to establish if, and how, trial teams report plans for retention at the design stage of clinical trials by examining a body of trial protocols. Our secondary aim is to compare the reported retention strategies with their evidence of effect. This will contribute to the evidence base for the PRioRiTy (Prioritising Retention in Randomised Controlled Trials) unanswered question “How should people who run trials plan for retention during their funding application and creation of the trial (protocol development)?” [12].

## Materials and methods

This scoping review has been conducted using the guidelines and framework outlined by the Joanna Briggs Institute [24], the most recent framework for scoping reviews [25–27]. This scoping review was reported using the Preferred Reporting Items for a Systematic Review and Meta-Analysis Protocols Extension for Scoping Reviews (PRISMA-ScR) [28] (Supplementary File 1). The protocol for this review is published in *Trials* and is also available in Supplementary File 2.

### Data sources and search strategy

The search strategy was developed in collaboration with a research librarian at University College Cork and is shown in Table 1. The following electronic databases were searched for relevant protocols, PubMed, Scopus, EMBASE, CINAHL (EBSCO), and Web of Science. The search was adapted as appropriate for each database using the software Polyglot [29] which translates search strategies across databases.

**Table 1:** PubMed search strategy

("randomised controlled trial"[Title/Abstract]) OR ("randomized controlled trial"[Title/Abstract])) OR ("randomised clinical trial"[Title/Abstract])) OR ("randomized clinical trial"[Title/Abstract])) OR ("randomized controlled trials as topic"[MeSH Terms])) AND ("protocol"[Title/Abstract])) OR ("clinical trial protocols as topic"[MeSH Terms])	

### Inclusion and exclusion criteria

We included the following: protocols for phase II, phase III and phase IV randomised controlled trials (RCTs), pilot and feasibility trials, and mixed methods studies that included a RCT element; protocols published between 2014 and 2019 (inclusive)—we chose this timeline to allow for sufficient time for the uptake of the SPIRIT guidelines published in 2013 [14] and then included a 6 year time-horizon, as this would provide a sufficient sample size; protocols of RCTs from any setting, that involved adults and/or children of any age, investigating any treatment/intervention type for any disease area/clinical specialty, investigating any comparator including placebo and examining any outcome; protocols for trials randomised at the cluster or individual level; protocols published in the English language. Excluded were as follows: non-protocol papers; protocols for non-randomised trials; protocols for quasi/partially randomised trials; protocols for single-arm trials; protocols for studies within a trial (SWATs); protocols for statistical analysis plans; protocols for phase 1 trials; protocols for process evaluations; protocols for economic evaluations; protocols for N-of-1 trials.

### Screening and selection process

**Fig. 1:**
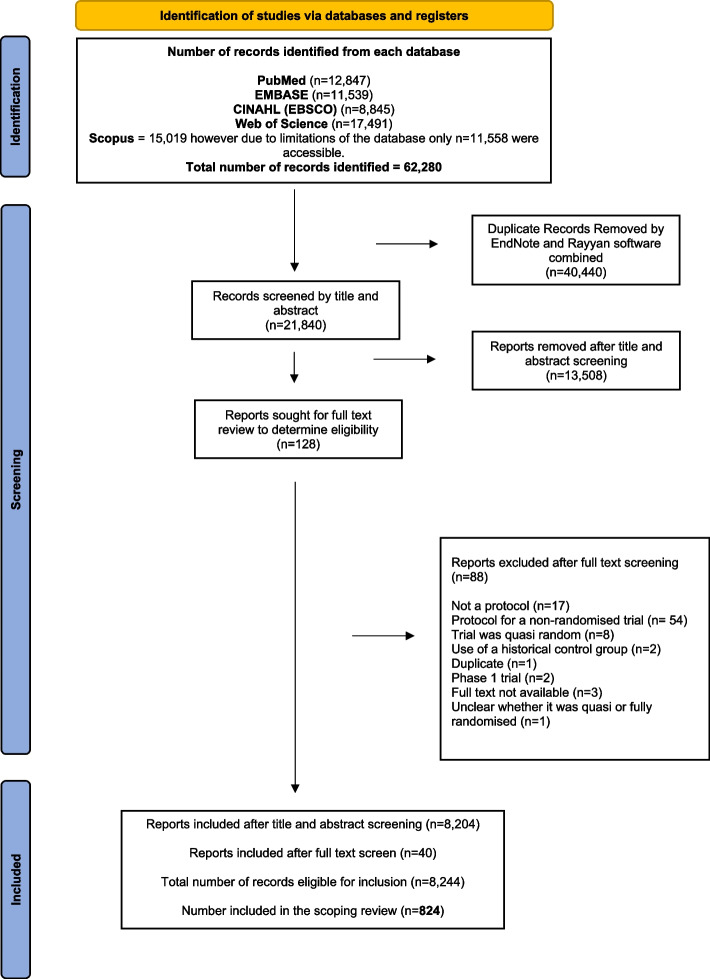
PRISMA flow diagram. Diagram showing the number of protocols included at each stage of the screening process of the scoping review

EM imported titles and abstracts of all electronically sourced search results to EndNote, grouping results separately for each database. Duplicates were removed and the remaining results were exported to Rayyan QCRI software for screening. The screening process involved two reviewers (EM and FS). EM independently screened all titles and abstracts. FS screened a random selection of 10% of the overall search output, this random 10% was selected using a random number generator. Where disagreement arose, a third reviewer KG was consulted, and when necessary, full protocol texts were obtained to determine eligibility. We set ourselves a target of 10% of the eligible protocols (*n* = 8244). We wanted a sample that was large enough to say something meaningful, but small enough to facilitate completion. Ten percent (*n* = 824) seemed a reasonable compromise between size and feasibility. See Fig. 1 for The PRISMA flow diagram.

### Data management and data extraction process

The full list of extracted variables, discussed and agreed upon by all authors, are outlined in the protocol (Supplementary File 2). The data extraction form was piloted by EM using a sample of 10 protocols and was reviewed by FS and KG to ensure the variables extracted best met the objectives of the scoping review. Data extraction was performed by EM and a random sample (10%) of the protocols was selected by FS using a random number generator, and checked to ensure consistency and improve the reliability of the data extraction process. All extracted information was entered into a Microsoft Excel file.

For the purposes of this scoping review, we defined a retention strategy as an action/activity that is conducted, in addition to usual follow-up procedures, with the purpose of retaining participants in a trial, reducing missing data or improving data completeness. We were not concerned with extracting information regarding activities to improve adherence or compliance to an intervention.

The outcome of interest was adherence to SPIRIT item 18b, “Plans to promote participant retention and complete follow-up including list of any outcome data to be collected for participants who *discontinue or deviate* from intervention protocols” ([14]:3). We divided this statement into three categories; 18b(i) “plans to promote participant retention”, 18b(ii) “plans to complete follow-up including list of any outcome data to be collected for participants who *discontinue* from intervention protocols” and 18b(iii) “plans to complete follow-up including list of any outcome data to be collected for participants who *deviate* from intervention protocols”. For 18b(i), we defined this as *proactive plans* outlined in the protocol that aim to actively promote participant retention in the trial. For 18b(ii) and 18b(iii), we defined these as *reactive plans* outlined by the trial team to complete outcome data collection and to complete follow-up of participants who have withdrawn/discontinued or deviated from the intervention protocols.

Regardless of whether the protocol reported using the SPIRIT guidelines in the protocol, we analysed all protocols for information that we could map to SPIRIT item 18b “Plans to promote participant retention and complete follow-up including list of any outcome data to be collected for participants who discontinue or deviate from intervention protocols” ([14]:3). Which as described above we divided into three categories.

### Data synthesis

We produced descriptive statistics on the characteristics of the trial protocols and on those adhering to SPIRIT item 18b. A narrative synthesis of the retention strategies was also conducted. The Guidance on the Conduct of Narrative Synthesis in Systematic Reviews were referred to during this process [30]. The retention strategies were coded by EM based on the type of retention strategy, i.e., reminders, prompts, monetary incentives. We mapped each strategy to the ORRCA (Online Resource for Research in Clinical triAls) retention domains [31] (ORRCA_Retention_Domains.pdf) as has previously been done in the Cochrane systematic review of strategies to improve retention in randomised trials [1]. The data items were mapped to 16 out of the 44 ORRCA domains, the most popular category being “B. Participants”. A total breakdown of the number of protocols mapped to each ORRCA domain can be seen in Table 4 along with example quotes that were mapped to each domain.

When it was unclear which ORRCA domain, a retention strategy mapped to FS and KG were consulted and a joint decision was made. Fifty of 307 data items were consulted upon. We made the following assumptions when mapping the retention strategies.

1. Following the approach taken by the Cochrane review [1], ORRCA domain “B1 Reminders” was divided into reminders (sent after a missed data collection time point) and prompts (sent before the data collection time point). In some cases, it was not clear if the strategy was intended as a reminder or prompt therefore based on the wording of the surrounding text we made a judgement call as to whether it was a reminder or prompt. If it was still unclear from this and the strategy included the word reminder/prompt, we mapped it to ORRCA as such.
2. When a protocol outlined the use of more than one retention strategy, we created a new category called “combined strategies”. We have detailed the most commonly combined strategies in Table 4.
3. Regarding monetary and non-monetary compensation for participants, we made the assumption that all monetary compensation functioned as incentives rather than rewards. Our reasoning for this is that ethically, patient information leaflets must disclose to participant information about receiving monetary compensation [20]. This prior knowledge of receiving monetary compensation means the compensation functions as an incentive rather than a reward.
4. We further classified monetary and non-monetary incentives as either conditional (based on the participant completing a task) or non-conditional (not based on participants completing a task).

Since we included pilot and feasibility trial protocols, we conducted a sub-group analysis of the retention strategies outlined in these protocols. The results from the pilot and feasibility protocols will be reported separately (“Analysis of pilot and feasibility trial protocols”). All other protocols that were not pilot and feasibility trials are included in what we will refer to as RCT protocols throughout the paper and Supplementary File 3.

## Results

### Protocol characteristics

Table 2 displays the characteristics of the protocols included in our analysis. In summary, of the 824 protocols included in the 6-year period: 26.6% (*n* = 219) were published in 2019; most trials were non-commercial, i.e. publicly funded trials that did not receive funding/donations from private for-profit companies (80.8%, *n* = 666); tested non-drug interventions such as diet, exercise, therapy, and educational interventions (72%, *n* = 593). Individually randomised designs dominated (84%, *n* = 692). Thirty-point-three percent (*n* = 250) of protocols were for trials conducted in vulnerable populations and 22% (*n* = 181) were for trials conducted among populations consisting of both vulnerable and non-vulnerable individuals. Vulnerable populations were defined by this reviews’ authors via local ethics committee [32] and ICH GCP definitions [20] these included; infants and children aged 17 and under, pregnant women, institutionalised individuals, adults aged 60 and over, critically ill patients not able to consent for themselves, homeless individuals and refugees, see Table 2 for full definition of all included populations. The protocols covered a wide range of clinical specialties, 38 in total, including oncology, musculoskeletal diseases, cardiology, neurology, nephrology, and obstetrics and gynaecology. The topic of public health was the most common (15.9%, *n* = 131). This category included trials evaluating interventions targeting for example physical activity, nutrition, smoking cessation, alcohol/drug misuse, gambling, obesity, sleep disorders, and family planning and contraception.

**Table 2:** Protocol characteristics (total protocols = 824)

**Year of publication**	**Number of protocols (*n*, %)**
2014	85 (10.3%)
2015	108 (13.1%)
2016	125 (15.2%)
2017	126 (15.3%)
2018	161 (19.5%)
2019	219 (26.6%)
**Level of randomisation**	
Cluster RCTs	132 (16%)
Individually randomised RCTs	692 (84%)
**Funding type**	
Commercial trial^a^	98 (11.9%)
Non-commercial trial	666 (80.8%)
No funding	27 (3.3%)
Unclear—no information provided	33 (4%)
**Type of intervention**	
Non-drug trial	593 (72%)
Drug trial	138 (16.7%)
Mix of intervention types	23 (2.8%)
Surgical trial	55 (6.7%)
Medical device trial	15 (1.8%)
**Patient population**^**b**^	**Number of protocols**
Vulnerable populations	250 (30.3%)
Mix of vulnerable and non-vulnerable populations	181 (22%)
Not vulnerable	73 (8.9%)
Unclear	320 (38.8%)
**Planned sample size**^**c**^ **Individual level randomisation (*n* = 692)**	**Number of protocols**
100 participants or less	222 (32.1%)
101–200 participants	175 (25.3%)
201–300 participants	84 (12.1%)
301–400 participants	58 (8.4%)
401–500 participants	19 (2.7%)
501 participants and greater	121 (17.5%)
Overlap of categories	2 (0.3%)
Unclear from protocol	11 (1.6%)
**Planned sample size**^**d**^ **Cluster trials (*n* = 132)**	**Number of protocols**
100 clusters or less	110 (83.3%)
101–200 clusters	8 (6.1%)
201–300 clusters	4 (3%)
301–400 clusters	
401–500 clusters	
501 clusters and greater	1 (0.8%)
Unclear from protocol	9 protocols (6.8%) – cluster size unclear but provided the participant size in 7 protocols • 1202 participants • 382 participants • 426 participants • 342 participants • 90 participants • 300 participants • 600 participants
**Clinical Specialty**^**e**^	**Number of protocols**
Public Health	131 (15.9%)
Musculoskeletal	80 (9.7%)
Oncology	77 (9.3%)
Mental Health	74 (9%)
Cardiology	74 (9%)
Obstetrics and Gynaecology	65 (7.9%)
Neurology	62 (7.5%)
Diabetes and Endocrinology	35 (4.2%)
Respiratory	33 (4%)
Sexual Health and STIs	30 (3.6%)
Nephrology	19 (2.3%)
Vascular diseases	19 (2.3%)
Gastroenterology	17 (2.1%)
Paediatrics	12 (1.5%)
Surgery and Anaesthesia	11 (1.3%)
Dental health	10 (1.2%)
Haematology	8 (1%)
Infectious Disease	8 (1%)
Intensive care	7 (0.8%)
Ophthalmology	7 (0.8%)
Hepatology	6 (0.7%)
Otology	6 (0.7%)
Autoimmune diseases	6 (0.7%)
Emergency care	4 (0.5%)
Palliative care	3 (0.4%)
Otolaryngology	3 (0.4%)
Dermatology	3 (0.4%)
Genetics	3 (0.4%)
Intellectual Disabilities	2 (0.2%)
Pathology	1 (0.1%)
Rehabilitation	1 (0.1%)
Trial Methods	1 (0.1%)
Secondary care	1 (0.1%)
Primary care	1 (0.1%)
Pharmacy care	1 (0.1%)
Geriatric medicine	1 (0.1%)
Orthopaedics	1 (0.1%)
Appendicitis	1 (0.1%)
**Patient reported primary outcome**	**Number of protocols**
Yes	298 (36.2%)
Partly^f^	78 (9.5%)
No	440 (53.4%)
Unclear from protocol	8 (1%)
**Number of follow-up assessments**	**Number of protocols**
1 follow-up assessment	124 (15%)
2 follow-up assessments	238 (28.9%)
3 follow-up assessments	156 (18.9%)
4 follow-up assessments	105 (12.7%)
5 follow-up assessments	34 (4.1%)
6 or more follow-up assessments	106 (12.9%)
Unclear from protocol	61 (7.4%)
**Follow-up method for data collection**	**Number of protocols**
In person clinic visit	290 (35.2%)
Postal questionnaire	13 (1.6%)
Electronic questionnaire /online assessment	49 (5.9%)
Telephone call	24 (2.9%)
Via patient records or databases^g^	25 (3%)
Home visits/visits to site outside the clinic by researcher	37 (4.5%)
A combination of follow-up methods	326 (39.6%)
All data collected whilst the participant is in the hospital	36 (4.4%)
Unclear from protocol	24 (2.9%)
**Routine data sources for data collection**^**h**^	
Yes	164 (19.9%)
No	660 (80.1%)
**Trial type**	
Pilot or feasibility trial	102 (12.4%)
RCTs	722 (87.6%)
**RCT protocols reported using SPIRIT guidelines**	
Yes	253 (35%)
No	469 (65%)
**Pilot and feasibility protocols reported using SPIRIT guidelines**	
Yes	35 (34.3%)
No	67 (65.7%)

^a^Commerial trials were defined as a trial that has any type of funding or donation from a private for-profit company/organisation for example partly funded by pharma or product provided by a commercial company was classified as a commercial trial

^b^Vulnerable populations were defined by this reviews’ authors via local ethics committee definition [32] and ICH GCP definition [20] these included; infants and children aged 17 years and under, pregnant women, institutionalised individuals (prisoners, in nursing homes, mental health institutions), critically ill/ICU patients/patients on ventilators unable to provide consent so deferred consent is gained, where stated in the protocol deferred consent is obtained, adults aged 60 and over, participants with learning disabilities, suffers of dementia, adults with terminal illness, homeless individuals and refugees, adults with mental illness, and members of the armed forces and medical/nursing/dental/pharmacy students where there is a hierarchy in the trial that would influence the decision to take part voluntarily

^c^The sample size groupings contain protocols that stated they would recruit “at least” or a “minimum (number) of” participants for example if a protocol stated they would recruit at least 80 participants this has been grouped into category 1. “100 participants or less”. For dyad pairs, these have been grouped in terms of total number of participants for example 100 participants and their dyad, i.e. 200 participants would be grouped in category 2. “101–200 participants”

^d^The sample size groupings contain protocols that stated they would recruit “at least” or a “minimum (number) of” clusters for example if a protocol stated they would recruit at least 80 clusters this has been grouped into category 1. “100 clusters or less”

^e^Categories were based on clinical specialty for example surgery for cancer was classed under “Oncology” rather than “Surgery and Anaesthesia”, only surgeries or anaesthetic procedures for non-specific clinical area/none of the clinical specialty categories listed above were grouped under “Surgery and Anaesthesia” for example “elective non-cardiac surgery”. Similarly, “Paediatrics” only contains paediatric trials that did not involve a clinical specialty area listed above, for example “Chronic Fatigue Syndrome” was include in “Paediatrics” whereas “Children younger than 5 years of age with acute gastroenteritis” was grouped into “Gastroenterology”

^f^Partly patient reported means aspects of the primary outcome were reported by the patient and other aspects were not

^g^In this category, participants are not directly followed up, all follow-up is via a database/registry/routine data source

^h^In this category, routine data sources were used for outcome data/follow-up data/demographic data on participants, these routine sources include patient records, registries, hospital databases and medical records

### Compliance with the SPIRIT 2013 Statement

Table 3 reports the key findings relevant to the use of the applicable retention items from the SPIRIT 2013 statement for RCT protocols (*n* = 722). We report separately on the pilot and feasibility protocols (*n* = 102). (A more detailed breakdown is provided in Supplementary File 3).

Of the 35% (*n* = 253) of RCT protocols that reported using the SPIRIT guidelines when developing the protocol, 9.5% (*n* = 24) fully complied and included all aspects of item 18b (18b(i) and 18b(ii) and/or 18b(iii)), and 41.5% (*n* = 105) included item 18b(i) “plans to promote participant retention” (proactive rather than reactive plans).

**Table 3:** Key SPIRIT 2013 statement results^a^

**Reported use of the SPIRIT guidelines**	**Number of RCT protocols**
Yes	**253 (35%)**
No	469 (65%)
**Reported using the SPIRIT guidelines and reported all aspects of item 18b (18b(i) and 18b(ii) and/or 18b(iii)) – “**Plans to promote participant retention and complete follow-up, including list of any outcome data to be collected for participants who discontinue or deviate from intervention protocols” ([14]:3)	
Yes	**24 protocols out of the 253 that reported using SPIRIT (9.5%)**
No	229 (90.5%)
**Reported using the SPIRIT guidelines (n = 253) and reported item 18b(i)** “Plans to promote participant retention”	**Number of RCT protocols**
Yes	**105 protocols out of the 253 that reported using SPIRIT (41.5%)**
No	148 protocols (58.5%)
**RCT protocol SPIRIT item 18b figures, regardless of reporting SPIRIT guidelines in the protocol, i.e. information mapped to SPIRIT item 18b**	
**Reported all aspects of item 18b (18b(i) and 18b(ii) and/or 18b(iii))—“**Plans to promote participant retention and complete follow-up, including list of any outcome data to be collected for participants who *discontinue or deviate* from intervention protocols” ([14]:3)	**Number of protocols**
Yes	**53 protocols out of the total (*n* = 722) (7.3%)**
No	669 protocols (92.7%)
**Reported item 18b(i)** “Plans to promote participant retention" (out of the total 722 RCT protocols)	
Yes	**266 (36.8%)**
No	456 (63.2%)

^a^Excludes pilot and feasibility protocol data (See Supplementary File 3 for a full breakdown of SPIRIT results)

### Plans to promote participant retention

Of the RCT protocols (*n* = 722) regardless of reporting using the SPIRIT guidelines, 7.3% (*n* = 53) included all aspects of item 18b (18b(i) and 18b(ii) and/or 18b(iii)), “plans to promote participant retention and complete follow-up, including list of any outcome data to be collected from participants who *discontinue or deviate* from intervention protocols” ([14]:3).

#### SPIRIT item 18b(i)

SPIRIT item 18b(i) “plans to promote participant retention” (proactive plans) was included in 36.8% (*n* = 266) of the RCT protocols, regardless of whether they reported using SPIRIT guidelines or not. The most common retention strategy was the use of “combined strategies” used in 48.1% of protocols (*n* = 128). The joint most popular combined retention strategies were the use of “reminders and data collection location and method” (e.g. use of return postage such as pre-paid stamped return envelopes, options of home visits/telephone/postal data collection versus clinic visits), and “reminders and monetary incentives”. The median number of retention strategies used in a singular protocol was 3. The highest number of strategies reported in any one protocol was 9.

In terms of individual retention strategies, the most common was “reminders” (14.7%, *n* = 39) followed by “monetary incentives-conditional” (10.2%, *n* = 27). Some of the least popular methods included “maintaining staff engagement” (0.4%, *n* = 1), and “monetary incentives – unconditional” (0.4%, *n* = 1).

Table 4 summarises the ORRCA domains mentioned in the protocols along with sample quotes from protocols. The most frequently used combined strategies are provided at the bottom of Table 4. A full list of all combinations of combined retention strategies can be found in Supplementary File 3.

**Table 4:** Plans to proactively promote participant retention (*n* = 266, 36.8%)^a^

**ORRCA Domains**	**Number (%)**	**Examples of quotes from RCT protocols**
**A. Data collection**		
**A2. Data collection frequency and timing**	**1 (0.4%)**	Participants will be monitored monthly for signs or symptoms of adverse effects. One month later, the participants will be reviewed in the clinic and a possible side effect check list will be conducted. **To prevent attrition and to assess adherence to treatment, telephone interviews will be conducted again at 2 months from administration of the drug**
**A3. Data collection location and method**	**12 (4.5%)**	The survey will be available online via qualtrics (https://www.qualtrics.com/) with a direct link sent to participants. *For those who prefer a hard copy, it will be posted with a return envelope*
		If participants in either group miss their scheduled visit, and it cannot be rescheduled within 4 weeks of their prior visit, the PC clinician may conduct the visit through telephone within seven days from the missed visit
**A5. Data collection during routine care**	**4 (1.5%)**	To minimise loss to follow-up, assessments are timed to coincide with routine clinical follow-up
		The primary outcome point will be collected at the final outpatient clinic appointment and, as such, it is anticipated that missing data for the primary outcome will be low
**B. Participants**		
**B1. Reminders (Including repeat contacting of participants via phone, post, email etc.)**	**39 (14.7%) – reminders**	**Reminders** Participants who do not complete and return the study questionnaires in the specified time period will be contacted by the research team via telephone or email, as a reminder about the study
		Another potential limitation for this trial is the attrition rate. It is possible to have a high rate of participant dropout and subsequently a significant loss of data. Therefore, reminders via telephone contact, email, and SMS will be used
**Prompts**	**17 (6.4%) – prompts**	**Prompts** The researcher will send reminder calls 3 days ***in advance*** to promote retention
		To promote participant retention, we plan training sessions in consultation with the participants and inform participants timely about the entire training schedule and the assessments
**Unclear**	**2 (0.8%)**	**Unclear** Phone or email reminders for completion of follow-up questionnaires were performed with phone or email prompts based on the tailored design method proposed by Dillman et al
		To optimise follow-up, multiple attempts will be made to contact participants including contacting their referring doctor and at a minimum we will aim to record vital status for all participants
**B2. Monetary incentives – direct cash provided to participants/gift vouchers, prizes that are monetary**		**Conditional monetary incentives** All families receive remuneration for their time: US $100 for completing the baseline assessment and US $100 for completing each of the 3 follow-up assessments, for a total of $400. Families can also earn a US $50 bonus if they complete all 4 assessments
**Conditional Incentives**	**27 (10.2%)**	To maximise participation and follow-up rates, we offered patients $20 for completing each questionnaire
**Unconditional incentives**	**1 (0.4%)**	**Unconditional monetary incentives** We recognise the importance of participant retention and will offer a voucher of £10 at recruitment
**B3. Non-monetary incentives –entry to raffles for prizes that are non-monetary, completion of trial certificates, offering the controls the intervention at the end of the trial**		**Conditional non-monetary incentives** The success of the intervention is strongly dependent on enjoyment and active participation. In order to motivate children’s active participation, the staff will use several strategies to celebrate success achieving the proposed objectives in both the healthy lifestyle education and the exercise programmes: celebrate and recognise their efforts, reward with smiley emoticons, etc. Children who complete the programme successfully will be rewarded with a certificate of completion
**Conditional incentives**	**3 (1.1%)**	Moreover, patients who complete the intervention will have an 8-h nutrition education programme for free
**Unconditional Incentives**	**4 (1.5%)**	**Unconditional non-monetary incentives** The primary purpose of employing an attention control intervention is to limit principals’ and teachers’ disappointment at not receiving the iPLAY intervention, thereby increasing participation during data collection at the post-intervention and maintenance phases To minimise loss to follow-up, all controls will be offered the intervention at study end
**B4. Maintaining participant engagement**	**14 (5.3%)**	Once a participant is included, every reasonable effort is made to prevent attrition through the entire study period. In addition to the planned visits, all participants have, during the last 2 years, received two letters in connection with milestones and holidays. Distribution of letters will continue throughout the entire study period
		Personal data will be used to contact the participant, to thank them for participating in the study, to facilitate the follow-ups at 6 months and 2 years of age, to co-ordinate the follow-ups and to disseminate the results of the study to participants
**B7. Supporting participation**	**7 (2.6%)**	Participants will be advised at the initial trial enrollment meeting to carefully consider the required investment of time and effort involved in the current project. This will be undertaken to minimise any negative impact associated with loss to follow-up, and consequently likely withdrawal can be made prior to randomisation
		The CRA will explain the study to these patients using comprehensive ethics committee-approved documents and patients will be given opportunity to ask questions and receive further information. This process is to ensure participants are fully informed of the possible burden of appointments and data collection on their time and to enhance retention and reduce loss to follow-up
**B8. Contact information**	**3 (1.1%)**	Minimising attrition in follow-up assessments is vital to ensure the success of this trial. The at-risk and transient nature of the target population makes this task more difficult. To mitigate this, participants will provide a collateral person who can be contacted. Participants will not be precluded from taking part in the study if they are not comfortable giving the details of collateral persons
		To increase follow-up data collection, clinicians will collect contact telephone numbers for the participant and at least two family members or caregivers
**B12. Motivations and experience**	**1 (0.4%)**	According to Danish research ethics legislation, we will inform the participants about their rights as voluntary subjects in a scientific trial and interview them about their motivation for participation. We do this to make participants consider participation thoroughly to diminish the likelihood of their dropping out
**C. Sites and Site Staff**		
**C4. Maintaining staff engagement**	**1 (0.4%)**	News letters from the project were regularly sent to all PHCC managers and RCs at both intervention and control centres
**E. Study Design**		
**E1. Choice of study outcomes**	**1 (0.4%)**	PCOMS could also reduce the number of dropouts and/or increased patient satisfaction, all leading to cost reduction
**E4. Randomisation method**	**1 (0.4%)**	Subjects are randomised on arrival in the operating theatre to minimise the risk of dropouts after randomisation
**Combined strategies**	**128 (48.1%)**	
**Protocols with multiple retention strategies (*n* = 128 (48.1%))** **Top most common combined strategies to proactively promote retention**		
**Strategy**		**Number of protocols (%)**
	• Reminder • Data collection location and method	**6 (4.7%)** **Example of quote** Participants can choose to complete the questionnaires online or by using a paper questionnaire. Non-respondents will be contacted by telephone within 2 weeks. If they do not respond to this reminder, they will be sent a reminder letter within 2 weeks
	• Reminder • Monetary incentive	**6 (4.7%)** 4 of the these were conditional monetary incentives 2 were unclear if they were conditional or unconditional monetary incentives
		**Example of quote** Participants will receive up to three reminder emails if they do not complete the research questionnaires within the allocated time frame. If the questionnaires are still not completed participants will be offered $A20 to complete it. Participants who comply with all study procedures will receive $A50
	• Prompt • Monetary incentive	**5 (3.9%)** All conditional incentives
	• Monetary incentives • Data collection location and method • Reminder	**5 (3.9%)** 4 of these were conditional monetary incentives 1 was an unconditional monetary incentive
	• Monetary incentives • Supporting participation	**4 (3.1%)** 3 of these were conditional monetary incentives For 1 it was unclear whether it was a condition or unconditional monetary incentive
	• Supporting participation • Maintaining participant engagement	**4 (3.1%)**
	• Prompt • Reminder • Monetary incentive	**4 (3.1%)** All conditional monetary incentives
	• Prompt • Reminder	**4 (3.1%)**
	• Reminders • Maintaining participant engagement	**3 (2.3%)**
	• Non-monetary incentive • Monetary incentive	**3 (2.3%)** 2 were both conditional for both types of incentives 1 was a conditional monetary incentive combined with a non-monetary incentive but it was unclear if it was conditional or unconditional
	• Monetary incentives • Data collection location and method	**3 (2.3%)** All conditional monetary incentives
	• Prompt • Maintaining participant engagement • Monetary incentive	**3 (2.3%)** 2 were conditional monetary incentives 1 included both a conditional and unconditional monetary incentive

^a^Excludes pilot and feasibility protocol data

#### SPIRIT items 18b(ii) and 18b(iii)

A combined total of 13.7% (*n* = 99) of the 722 RCT protocols considered a reactive plan to collecting outcome data (SPIRIT item 18(ii) and/or item 18b(iii)), “plans to complete follow-up including list of any outcome data to be collected for participants who *discontinue and/or deviate* from intervention protocols”, regardless of reporting using SPIRIT guidelines or not. No strategy actively targeted those that might withdraw from the trial, i.e. strategies typically employed in 18b(ii) and 18b(iii) were seeking consent early in the trial for continued use of the data if they withdrew or deviated from the protocol. A full breakdown of results can be found in Supplementary File 3.

### Analysis of pilot and feasibility trial protocols

Of the 824 trial protocols, 12.4% (*n* = 102) were for pilot and feasibility trials. Of these, 34.3% (*n* = 35) used the SPIRIT statement during protocol development. Of those 35 protocols, 11.4% (*n* = 4) included all three parts of SPIRIT item 18b (18b(i) and 18b(ii) and/or 18b(iii)) [14].

Overall, 40.2% (*n* = 41/102) included item 18b(i) “plans to promote participant retention”, a proactive plan to promote retention, regardless of whether they reported using SPIRIT guidelines or not. A combined total of 14.7% (*n* = 15) of protocols included a reactive plan (SPIRIT items 18b(ii) and/or 18b(iii)) “plans to complete follow-up for those who *discontinue and/or deviate* from the intervention protocols”. A total of 6.9% (*n* = 7) protocols reported all aspects of SPIRIT item 18b (18b(i) and 18b(ii) and/or 18b(iii)) [14].

A total breakdown of the number of pilot and feasibility protocols mapped to each ORRCA domain can be seen in Table 5 along with examples of quotes that were mapped to each domain. The top combined strategies are also shown in Table 5. See Supplementary File 3 for full details of all combinations of combined retention strategies.

**Table 5:** Analysis of Pilot and Feasibility trial protocols

**Pilot and feasibility trial protocols with a retention strategy (*n* = 41, 40.2%)**		
**ORRCA Domain**	**Number of protocols (%)**	**Examples of quotes from the protocols**
**Data collection**		
A3. Data collection location and method	2 (4.9%)	Patients who have consented to participate receive a questionnaire and pre-paid addressed envelope
**B. Participants**		
B1. Reminders (including repeat contacting of participants via phone, post, email)	3 protocols (7.3%)	**Reminder** Patients who were allocated to Fatigue Information Sheet only, will be asked about their experience of reading the Fatigue Information Sheet. A postal reminder will be sent to non-responders 2 and 4 weeks after the 7-day response period has ended, utilising the Reminder Letter and/or a telephone call. Six and 12 months post randomisation, two more outcome booklets will be sent respectively, with two postal reminders and/or telephone calls for non-responders after 2 and 4 weeks
Prompts	2 protocols (4.9%)	**Prompt** All clinical outcomes, except for limb circumference, will be collected via self-report questionnaires. Reminder emails and/or calls will be sent out by the Research Assistant *prior to each follow-up assessment* at week 5 and week 11
B2. Monetary incentives – direct cash provided to participants/gift vouchers, prizes that are monetary	9 protocols (22%)	Participants in both arms will receive a modest monetary compensation of $30CAD each time they meet with the research assistant for data collection every 3 months for an expected time of 1 h (five times total). This amount is seen as a token of appreciation yet non-coercive
Conditional incentives		
B3. Non-monetary incentives—entry to raffles for prizes that are non-monetary, completion of trial certificates, offering the controls the intervention at the end of the trial	1 protocol (2.4%)	To prevent attrition, condensed WheelSeeU training or iWheel information is offered to all participants at the end of the study
Unconditional incentives		
B4. Maintaining participant engagement	1 protocol (2.4%)	All reasonable efforts, within the CRF local standard operating procedure, will be made to ensure optimum participant engagement and to reduce study attrition
B7. Supporting participation	1 protocol (2.4%)	The follow-up appointment will be arranged during the baseline meeting, at a time convenient to participants, and will take place in a clinic at the hospital
B8. Contact information	2 protocol (4.9%)	Participants are asked to give their own details as well as those of a family member or friend in case it is difficult to contact them directly
**C. Sites and site staff**		
C6. Trial site factors	1 protocol (2.4%)	The intention of conducting the study within the neighbourhood of the participant is to facilitate the transferability of training and to improve the ecological validity. In addition, training in the community aims to reduce participant burden of travelling to our research site, and to improve adherence
** Combined strategies**	19 protocols (46.3%)	
**Top most common combined retention strategies;**		
• Monetary incentives – conditional • Prompt	2 (10.5%)	Study participants will be contacted at 3 months by a study researcher to confirm contact details and as a reminder about the 6-month assessment. A follow-up interview will then be scheduled for 6 months after randomisation. All participants will be offered a £20 honorarium following completion of the 6-month follow-up interview
• Monetary incentives – conditional incentive • Supporting participation • Data collection location and method	2 (10.5%)	Women from both trial groups will be asked to attend an appointment with a research midwife to be weighed either at the study site or at their home at 6 and 12 months. Travel costs and £10 Love2Shop voucher to thank women for their time will be offered. Follow-up appointments will be offered at weekends and week days, with the option to complete questionnaires at these appointments
• Data collection location and method • Reminder	2 (10.5%)	The follow-up questionnaires are posted to participants with a reply paid envelope. The protocol for following up questionnaires begins with a 2-week waiting period (from postage date) and four phone calls over 7 days if it is not received within this time. Should phone contact be unsuccessful, research staff contact the recruiting site to check the situation of the patient (e.g. patient death). If the patient’s situation has changed, research staff review carer’s eligibility in collaboration with clinical staff at the site. If the patient’s situation is unchanged, a replacement questionnaire is sent and the same waiting period and phone call schedule are followed. Participants are withdrawn if contact is not made after this second waiting period

### Evidence to support the use of retention strategies

Table 6 displays the top 10 most popular retention strategies identified in this review mapped to the evidence of their effectiveness from the Cochrane review of strategies to improve retention in randomised controlled trials [1]. The Cochrane review defines these strategies as “those designed to generate maximum data return or compliance and follow-up procedures that aim to collect data from participants” ([1]:22). The evidence to support the use of these strategies is either lacking entirely or in the majority of cases has a low-GRADE certainty rating. GRADE (Grading of Recommendations, Assessment, Development and Evaluations) is the most widely adopted tool for grading the quality of evidence and for making clinical practice recommendations and is endorsed by Cochrane.

**Table 6:** Most popular retention strategies compared against evidence for effectiveness

**Top 10 most popular retention strategies in the scoping review**^**a**^	**Evidence from the Cochrane Review** [1] **to support the use of the strategies found in the scoping review**
**Reminders (*n* = 39, 14.7%)**	**Reminders** Evidence to support the use of various types of reminders is very uncertain and may result in little or no difference to retention rates, the GRADE of evidence for such reminders is either low or very low. Only telephone reminders compared to postal reminders may result in a large increase in retention rates; however, the GRADE of evidence is low.
**Monetary incentives (conditional) (*n* = 27, 10.2%)**	**Monetary incentives** Monetary incentives compared to no incentive may increase retention but the GRADE of evidence is low. The addition of monetary incentives in all trial arms may favour the higher value incentive to increase retention but the GRADE of evidence is low.
	Addition of a monetary reward to both trial arms delivered either with the prenotification or with the reminder letter, probably leads to an increase in retention rates, the GRADE of evidence is moderate.
	Evidence regarding the use of other types of monetary incentives are very uncertain and may lead to little or no difference in retention rates, with the GRADE of evidence being low or very low.
**Prompts (*n* = 17, 6.4%)**	**Prompts** Evidence to support the use of prompts is very uncertain and may lead to little or no difference in terms of retention rates, GRADE of evidence is low or very low.
	Only prenotification cards vs no card and electronic prompts compared to electronic reminders looks to favour electronic reminders at increasing retention rates; however, the GRADE of evidence for both of these methods is low.
	Personalised prompts versus usual follow-up may reduce retention rates slightly but again the GRADE of evidence is low.
**Maintaining participant engagement (*n* = 14, 5.3%)**	The evidence to support the use of various strategies to maintain participant engagement with the hopes of improving retention is very uncertain and may lead to little or no improvement in retention rates, the GRADE of evidence is low or very low for these strategies.
	Including a newspaper article about the trial compared to no article may increase retention, similarly frequency of telephone contact comparing only at baseline to annual contact to contact only at baseline may increase retention but the GRADE of evidence for both strategies is low.
**Data collection location and method (*n* = 12, 4.5%)**	Evidence is very uncertain and may lead to little or no difference in retention regarding postal vs clinic follow-up and regarding telephone follow-up vs postal follow-up, evidence GRADE is very low.
	The use of first-class postage for outward mail versus second class postage may increase retention slightly, but the GRADE of evidence is low.
	Using free post versus second class stamp; high-priority mail stamp versus usual postage; and personal form all compared to usual postage practice for return postage may increase retention slightly but again the GRADE of evidence is low.
	The use of self-sampling kits (directly mailed or an invitation to order) probably increase retention, the GRADE of evidence is moderate.
**Supporting participation (*n* = 7, 2.6%)**	No evidence from the Cochrane review
**Data collection during routine care (*n* = 4, 1.5%)**	No evidence from the Cochrane review
**Non-monetary incentives (unconditional) (*n* = 4, 1.5%)**	Including a pen compared to no pen may increase retention slightly but the GRADE of evidence is low.
	The inclusion of a societal benefit messaged compared to usual follow-up may lead to little or no difference in retention rates, however the GRADE of evidence is low.
	The evidence to support the use of providing a certificate of appreciation compared to no certificate is very uncertain, and the GRADE of evidence is very low.
**Contact information (*n* = 3, 1.1%)**	No evidence from the Cochrane review
**Non-monetary incentives – (conditional) (*n* = 3, 1.1%)**	See above for evidence for non-monetary incentives

^a^Although the most common retention strategy in the review were the use of “combined strategies” used, we did not include this in the table as combined methods were not evaluated in the Cochrane Review [1]

## Discussion

The protocols included in this review covered a wide variety of clinical specialties, intervention types, sample sizes, patient populations, numbers and modes of participant follow-up. The overall reporting of the use of SPIRIT guidelines [14] during protocol development was low, with only 35% of RCT protocols and 34.3% of pilot and feasibility trial protocols reporting its use when developing the protocol. The SPIRIT guidelines were published in 2013 and taking this into consideration our search started in 2014 allowing a year for guideline uptake. Despite this, our results show the reporting of the use of SPIRIT guidelines when developing protocols is still low. Given the endorsement of SPIRIT by many journals such as BMJ, The Lancet, and JAMA, and by Biomed Central Journals, we believe this is a reporting issue rather than an implementation issue. Though the level of endorsement varies, either through general support for SPIRIT, encouraging protocol authors to use SPIRIT when developing protocols or explicitly requiring protocols to adhere to SPIRIT [33], which is seen in journals such as *Trials* and PLOS ONE, trialists are inevitably aware of SPIRIT, thus confirming our view that reporting of SPIRIT is poor, whilst implementation of SPIRIT is undoubtedly better than we were able to provide evidence for in this scoping review.

We were particularly interested in the reporting of item 18b which relates to trial retention. Adherence to this was quite low, suggesting that though trialists report using SPIRIT, reporting on retention is very poor. Of the 35% of RCT protocols that reported using SPIRIT, there was incomplete reporting of item 18b. Only 9.5% (*n* = 24) of these protocols fully complied and included all aspects of this item, “plans to promote participant retention and to complete follow-up, including list of any outcome data for participants who discontinue or deviate from intervention protocols” ([14]:3). The poor reporting of plans to promote participant retention in trial protocols could be because trial teams are initially worried about recruitment rates meaning retention is not a priority during planning but a reaction during conduct. It is also difficult to plan retention strategies when there is no evidence to support using most strategies [1], possibly lending itself into the issue of retention being considered after the fact. Additionally, strategies used to promote retention such as building relationships and maintaining rapport between trial staff and participants [23, 34] may not be reported in protocols as they may be considered more informal strategies [23] that may be difficult to plan, report and evaluate [34]. Furthermore, the lack of reporting poses issues for replication, trial teams may have plans to actively promote participant retention however due to poor reporting, these plans cannot be replicated for evaluation in the future.

Regardless of reporting using SPIRIT guidelines, out of the total 722 protocols for RCTs, only 36.8% reported a proactive plan to actively promote participant retention, meaning 63.2% of protocols did not consider proactively tackling the issue of retention during protocol development. Of the 102 pilot and feasibility protocols, only 40.2% outlined a proactive plan to promote participant retention. This lack of consideration for retention strategies during the design stage of the trial could be due to the emphasis on recruitment or other research priorities. Previous research shows there is still a stronger emphasis on recruitment more so than retention in trials [23, 35]. Trial staff believe reasons for this include funders and research networks place more emphasis on recruitment targets as trial performance is often based on recruitment rates [23]. Additionally, there are statistical methods used in trials to predict outcomes for individuals who have not been retained based on the available data for these participants [6], a possible factor as to why retention may not be considered as important as recruitment. This emphasis means that recruitment is incorporated into specific staff roles and trial teams may not be sufficiently informed about retention strategies [35]. Retention is a widespread issue of concern within trials [1, 10] and poor retention rates should not be a surprise to trial teams; therefore, trial teams should be considering retention strategies when they are designing the trial and developing the protocol.

Not considering retention during trial design and protocol development may lead to protocol amendments, getting further ethical approval, implementing the amendments may require more time and more personnel time. Additionally, not considering retention strategies from the outset can have budget implications as some of the most routinely used retention strategies by CTUs (clinical trial units) in the UK can be expensive to implement [3], the evidence to support their use is lacking [1] and few retention strategies show evidence of cost effectiveness [36]. Therefore, careful consideration and foreplaning is needed to ensure resources are utilised in the best way possible to yield the highest chances of successfully retaining trial participants. We acknowledge that trial teams may have planned retention strategies but failed to report them in the protocol. This lack of communication can lead to implementation issues if there is no clear plan outlined in the protocol document as trial staff use protocols for trial conduct throughout all stages of the trial [14]. A lack of information in the protocol also reduces transparency in trial conduct [14] and limits the replicability of retention strategies which has been recommended to investigate their effects on retention rates [1].

The use of “combined strategies” was the most popular among trial teams (of those that reported a retention strategy) in protocols for both RCTs and pilot and feasibility trials. This concurs with the Cochrane review evaluating strategies to improve retention in randomised trials [1]. Currently, the evidence to demonstrate that retention strategies are effective at retaining participants is either weak or entirely lacking with low to moderate GRADE ratings and no retention strategy has a high certainty GRADE rating to support their use [1]. Therefore, many trial teams are using strategies that may or may not improve retention rates, reinforcing the need for trial teams to plan, report in advance and evaluate the strategies used. This will help to generate evidence to determine which strategy to implement to maximise participant retention rates, whilst also weighing up the cost and resources required to implement the strategy. Implementing multiple strategies also needs further consideration in terms of evaluating their individual effectiveness, as this may be problematic if interaction effects between the different retention strategies are not considered.

Reducing the burden on participants to participate and to provide follow-up data is important in terms of retention [37] and identifying how best to retain participants will save time and trial costs [1]. Patient and public involvement (PPI) in research is important and varying levels of involvement exist. Sometimes PPI members are involved in one specific aspect of the trial or they can be involved for the trial duration [38]. PPI involvement [39] as well as involvement from healthcare professionals [40] during the initial stages of the trial development is important as it can help optimise the relevance of the research to the participant [39, 40] and once the trial is developed and the research question is decided it becomes harder for PPI members to influence key trial aspects [39, 41, 42]. Despite the importance of PPI involvement in the early stages of the trial such as trial development, there tends to be limited PPI input at this stage [42]. This review found a lack of PPI involvement in trial protocols that reported “plans to promote participant retention”. Therefore, we can only assume that PPI input was minimal at best. Thus, trial teams have lost an opportunity to ascertain if their chosen retention strategies are acceptable and suitable to their target population. This is another example of a chronic waste of participants’ time, and undoubtedly adds unnecessarily to trial costs. Another important note is that the majority of retention strategies in our scoping review were generic trial population level strategies and did not make recommendations about target groups within the trial for whom retention may be poorer. There is often also an overreliance on blanket approaches to improve retention with little evidence to support their use [43, 44]. Within trials there may be specific groups of individuals who are more likely to dropout of trials than others and trial teams may need to consider this when planning retention strategies to ensure the strategies they choose target these individuals who are at a higher risk of changes to participation status. One such group would be those participants who actively withdraw from a trial.

Plans to collect outcome data for those who withdraw/discontinue or deviate from the trial protocol (SPIRIT items 18b(ii) and 18b(iii)) were also lacking. There were no strategies that actively targeted withdrawers. Instead, the strategies were either passive—standard practice regarding continued use of collected data, or a more active plan asking participants for consent to continue data collection, despite discontinuation or deviation from the intervention protocol.

### Recommendations for future research

Going forward, trial teams need to consider plans to promote participant retention during protocol development, and these should be developed with PPI input. As part of this research programme, we will be conducting qualitative research to investigate why this currently does not seem to be the case, and to further delve into the nuances of these review findings. Retention strategies should be evidence-based strategies that are financially viable, operationalizable, implementable and, importantly, relevant for patients. It is also important for trial teams to consider the environmental sustainability of the retention strategies they choose to implement.

If existing evidence-based retention strategies are not suitable, or trial teams wish to use an alternative strategy, these should be evaluated alongside the trial or within the trial as a SWAT (study within a trial) to contribute to the evidence to support or not support their use. Most of the existing evidence is weak, or entirely lacking, regarding the effectiveness of retention strategies [1]. The Northern Ireland SWAT Repository (https://www.qub.ac.uk/sites/TheNorthernIrelandNetworkforTrialsMethodologyResearch/SWATSWARInformation/Repositories/SWATStore/) contains protocols for SWATs that have a retention focus and would provide the much needed evidence needed to decide if the most commonly used retention strategies are effective [45]. We would encourage all trial teams to look at this repository and utilise it. Additionally, the Cochrane review outlines specific priorities for the evaluation of retention strategies which we urge trial teams to take guidance from [1]. To ensure that resources are optimised to retain participants, we need evidence to guide the decision-making process when choosing retention strategies, without this evidence resources are potentially being wasted on strategies that may or may not improve retention rates in trials.

We recommend improved communication of plans to promote participant retention. It was difficult in some cases to distinguish between the use of reminders and prompts due to the language used in some descriptions. We direct trial teams to the ORRCA retention domains [31] and to the most recent Cochrane review of strategies to improve retention in trials, with no high certainty evidence of improvements on trial retention [1], to better communicate their retention strategies. This will assist the conduct of meta-analyses in the future.

Meaningful involvement of members from PPI groups and healthcare professionals [40] is important and valuable at the planning and design phase of a trial [38–40]. Currently, not only is there a lack of planning and/or communication of plans to promote participant retention in protocols, but of those that do report a retention strategy, there appears to be little input from PPI colleagues to indicate if these methods are suitable and acceptable to use among the target audience. We need the perspectives and opinions of these individuals to ensure that the strategies being planned are well received by the participants to have the best chance of success. In the UK the National Institute of Health Research now expects active PPI involvement in the research it funds [38, 46], but is it important this is not tokenistic [47]. The Health Research Board in Ireland also recommends working with PPI colleagues in the research it funds. We also direct trial teams to read Trial Forge Guidance 3 which is available as an open access document, to ensure they are taking steps to help recruit and retain individuals from under-served groups and that members of these groups are included in PPI groups [48].

### Strengths and limitations

The main strength of this review is the large sample size (*n* = 824) which includes a wide variety of trial protocols covering different clinical specialties and intervention types. This means that the results are generalizable, representative of RCT protocols and are relevant to a wide variety of trial teams and researchers.

There are some limitations in this review. As mentioned in the “Materials and methods” section, we had to make assumptions regarding some of the reported retention strategies due to a lack of detailed reporting in protocols. We assumed, based on standard ethics committees applications, that all monetary compensation would be disclosed to participants via patient information leaflets [20]. This prior knowledge means that all monetary compensation acted as a monetary incentive rather than a monetary reward. Due to the use of the words reminder and prompt interchangeably, we made assumptions based on the wording of the surrounding text indicating timing of delivery whether it was a reminder or prompt. Therefore, based on these assumptions, we may have misclassified certain retention strategies. We do not believe this has interfered with the overall findings and conclusions however we cannot state this for certain.

We are also aware that the published protocols in the review may not be the first iteration of the protocol, but it was not practical within the scope of our review to track down all versions of the trial protocols. However, due to excluding PsycINFO from our search which specifically specialises in behavioural and mental health trials, these trial protocols may be underrepresented in this review.

Whilst EM screened and data extracted all included protocols, 10% were double screened and double data extracted by FS, and a third reviewer (KG) was consulted where disagreements arose between EM and FS during these processes. This is a limitation as there is a higher possibility of error in the screening (missed protocols/incorrect inclusion of protocols) and the data extraction (relevant data not extracted) processes than if we had double screened and double data extracted all 824 included protocols. This may have impacted the results as relevant protocols and data may have been excluded; therefore, our results may be an underestimation of the reporting of retention strategies in trial protocols.

We also acknowledge that by the time this review is complete and published the timeline may seem out of date as it includes trials from 2014 to 2019 (inclusive), but we sought to establish if planning and reporting of retention plans occurs since the relevant SPIRIT 2013 guidelines were introduced and our inclusion criterion for a 6-year period post 2013 was suitable for that. We still recognise however that the findings may not as accurately reflect protocols written today.

## Conclusion

The purpose of our review was to establish if and how trial teams plan for retention at the design stage of clinical trials. Results show that trial teams often do not report plans to prospectively promote participant retention at the design stage of the trial, indicating that the SPIRIT 2013 guidelines item 18b is not being fully considered by trial teams. A greater focus on prospectively planning proactive of retention strategies may inform more suitable choice of strategies and may help lay the groundwork for improving retention rates throughout the course of the trial. Reporting these strategies in protocols also will increase replicability and transparency in trial conduct. Due to the widespread issue of poor retention in clinical trials, trial teams need to pay attention to retention.

### Supplementary Information

**Additional file 1. **

**Additional file 2. **

**Additional file 3. **

## Data Availability

The dataset created, used and analysed during this review is available from the authors on reasonable request.

## Abbreviations

ICH GCP: International Conference on Harmonisation of technical requirements for registration of pharmaceuticals for human use – Good Clinical Practice
FDA: U.S Food and Drug Administration
SPIRIT: Standard Protocol Items: Recommendations for Interventional Trials
PRioRity: Prioritising Retention in Randomised Controlled Trials
PRISMA-ScR: Preferred Reporting Items for a Systematic Review and Meta-Analysis Protocols Extension for Scoping Reviews
RCTs: Randomised controlled trial(s)
SWATs: Studies within a Trial
PRISMA: Transparent Reporting of Systematic Reviews and Meta-Analyses
ORRCA: Online Resource for Research in Clinical triAls
GRADE: Grading of Recommendations, Assessment, Development and Evaluations
CTUs: Clinical trial units
PPI: Patient and public involvement
