# Supplementary material for: How do trial teams plan for retention during the design stage of the trial? A scoping review
Source: Trials. 2023 Dec 4;24:784. doi: 10.1186/s13063-023-07775-2 (PMC10694955; doi:10.1186/s13063-023-07775-2)
Supplement: Supplementary file 3 — Additional file 3. [file 13063_2023_7775_MOESM3_ESM.docx]

**Supplementary File 3 – SPIRIT Information and a list of all combinations of retention strategies.**

**Table of Content Page**

SPIRIT Information breakdown – RCT protocols 2

SPIRIT Information breakdown – Pilot and Feasibility trial protocols 4

List of all ‘combined strategies’ – RCT protocols 6

List of all ‘combined strategies’ – Pilot and Feasibility trial protocols 14

Table 1 provides SPIRIT information breakdown for RCT protocols.

| **Table 1: SPIRIT 2013 Statement Results for RCT protocols (n=722 protocols)** | |
| --- | --- |
|  | |
| **Reported use of the SPIRIT guidelines** | **Number of RCT protocols (n, %)** |
| Yes | 253 (35%) |
| No | 469 (65%) |
|  |  |
| **Reported using the SPIRIT guidelines and reported all aspects of item 18b (18b(i) and 18b(ii) and/or 18b(iii)) – “**Plans to promote participant retention and complete follow-up, including list of any outcome data to be collected for participants who discontinue or deviate from intervention protocols” (1;3) |  |
| Yes | **24 protocols out of the 253 that reported using SPIRIT (9.5%)** |
| No | 229 (90.5%) |
|  |  |
|  |  |
| **Reported using the SPIRIT guidelines (n=253) and reported item 18b(i) “plan to promote participant retention”** | **Number of protocols (n, %)** |
| Yes | **105 protocols out of the 253 that reported using SPIRIT (41.5%)** |
| No | 148 protocols (58.5%) |
|  |  |
| **Reported using the SPIRIT guidelines (n=253) and reported item 18b(ii) and/or 18b(iii)** plans to “complete follow up including list of any outcome data to be collected for participants who **discontinue or deviate from protocol interventions** |  |
| Yes | **40 protocols out of the 253 that reported using the SPIRIT (15.8%)** |
| No | 213 protocols (84.2%) |
|  |  |
| **RCT protocol SPIRIT item 18b figures, regardless of reporting SPIRIT guidelines in the protocol i.e., information mapped to SPIRIT item 18b.** | |
|  | **Number of protocols (n, %)** |
| **Reported all aspects of item 18b (18b(i) and 18b(ii) and/or 18b(iii)) – “**Plans to promote participant retention and complete follow-up, including list of any outcome data to be collected for participants who discontinue or deviate from intervention protocols” (1;3) |  |
| Yes | **53 protocols out of the total (n=722) (7.3%)** |
| No | 669 protocols (92.7%) |
|  |  |
| **Reported item 18b(i)** “Plans to promote participant retention" (out of the total 722 protocols) |  |
| Yes | **266 (36.8%)** |
| No | 456 (63.2%) |
|  |  |
| **Reported item 18b(ii)** – Plans to complete follow up including list of any outcome data to be collected for participants who **discontinue from intervention protocols** |  |
| Yes | **85 (11.8%)** |
| No | 637 (88.2%) |
|  |  |
| **Reported item 18b(iii)** – Plans to complete follow up including list of any outcome data to be collected for participants who **deviate from intervention protocols** |  |
| Yes | **8 (1.1%)** |
| No | 714 (98.9%) |
|  |  |
| **Reported item 18b(ii) and 18b(iii)** – Plans to complete follow up including list of any outcome data for participants who **discontinue and deviate from intervention protocols** |  |
| Yes | **6 (0.8%)** |
| No | 716 (99.2%) |
|  |  |
| **Reported item 18b(ii) and/or 18b(iii)** Plans to complete follow up including list of any outcome data to be collected for participants who **discontinue and/or deviate from intervention protocols** |  |
| Yes | **99 protocols (13.7%)** |
| No | 623 protocols (86.3%) |
|  |  |
|  | |
| **Mention of PPI (Patient and Public Involvement) in relation to retention** | **Number of protocols (n, %)** |
| **For protocols with a retention strategy (n=266)** |  |
| Yes | 2 (0.8%) |
| Possible PPI involvement^1^ | 9 (3.4%) |
| No | 255 (95.9%) |
|  | |
| **Mention of cost associated with a retention strategy** | **Number of protocols (n, %)** |
| **For protocols with a retention strategy (n=266)** |  |
| Yes | 80^2^ (30.1%) |
| No | 186 (69.9%) |

*Excluding data from Pilot and Feasibility studies. ^1^Mention of PPI (patient and public involvement), where there was a plan to promote participant retention and PPI was mentioned but it was unclear if PPI members were involved in the designing and planning of the retention strategy specifically that was included as a “possible PPI involvement”. ^2^ 80 protocols mentioned a cost associated with the retention strategy however this was all related to the cost of the monetary incentive/compensation that was going to be used to promote participant retention. Not all monetary incentives mentioned cost.

Table 2 provides SPIRIT information for Pilot and Feasibility trial protocols.

| **Table 2: SPIRIT 2013 Statement Results for Pilot and Feasibility trial protocols (n=102)** | |
| --- | --- |
|  | |
| **Reported use of the SPIRIT guidelines** | **Number of protocols (n, %)** |
| Yes | 35 (34.3%) |
| No | 67 (65.7%) |
|  |  |
| **Reported using the SPIRIT guidelines and reported all aspects of item 18b (18b(i) and 18b(ii) and/or 18b(iii)) – “**Plan to promote participant retention and complete follow-up, including list of any outcome data to be collected for participants who discontinue or deviate from intervention protocols” (1;3) |  |
| Yes | **4 protocols out of the 35 that reported using SPIRIT (11.4%)** |
| No | 31 (88.6%) |
|  |  |
|  | |
| **Reported using the SPIRIT guidelines (n=35) and reported item 18b(i) “plan to promote participant retention”** | **Number of protocols (n, %)** |
| Yes | **15 protocols out of the 35 that reported using SPIRIT (42.9%)** |
| No | 20 protocols (57.1%) |
|  | |
| **Reported using the SPIRIT guidelines (n=35) and reported item 18b(ii) and/or 18b(iii)** plans to “complete follow up including list of any outcome data to be collected for participants who **discontinue or deviate from protocol interventions** |  |
| Yes | **7 protocols out of the 35 that reported using the SPIRIT (20%)** |
| No | 28 protocols (80%) |
|  | |
| **Pilot and Feasibility trial protocol figures, regardless of reporting SPIRIT guidelines in the protocol i.e., information mapped to SPIRIT item 18b.** | |
|  | **Number of protocols (n, %)** |
| **Reported all aspects of item 18b (18b(i) and 18b(ii) and/or 18b(iii)) – “**Plans to promote participant retention and complete follow-up, including list of any outcome data to be collected for participants who discontinue or deviate from intervention protocols” (1;3) |  |
| Yes | **7 protocols out of the total (6.9%)** |
| No | 95 protocols (93.1%) |
|  |  |
| **Reported item 18b(i) “Plans to promote participant retention"** |  |
| Yes | **41 (40.2%)** |
| No | 61 (59.8%) |
|  |  |
| **Reported item 18b(ii)** – Plans to complete follow up including list of any outcome data to be collected for participants who **discontinue from intervention protocols** |  |
| Yes | **13 (12.7%)** |
| No | 89 (87.3%) |
|  |  |
| **Reported item 18b(iii)** – Plans to complete follow up including list of any outcome data to be collected for participants who **deviate from intervention protocols** |  |
| Yes | **1 (1%)** |
| No | 101 (99%) |
|  |  |
| **Reported item 18b(ii) and 18b(iii)** – Plans to complete follow up including list of any outcome data for participants who **discontinue and deviate from intervention protocols** |  |
| Yes | **1 (1%)** |
| No | 101 (99%) |
|  |  |
| **Reported item 18b(ii) and/or 18b(iii)** Plans to complete follow up including list of any outcome data to be collected for participants who **discontinue and/or deviate from intervention protocols** | **Number of protocols (n, %)** |
| Yes | 15 protocols (14.7%) |
| No | 87 protocols (85.3%) |
|  |  |
|  | |
| **Mention of PPI (Patient and Public Involvement) in relation to retention** | **Number of protocols (n, %)** |
| **For protocols with a retention strategy (n=41)** |  |
| Yes | 0 |
| Possible PPI involvement^1^ | 7 (17.1%) |
| No | 34 (82.9%) |
|  | |
| **Mention of cost associated with a retention strategy** | **Number of protocols (n, %)** |
| **For protocols with a retention strategy (n=41)** |  |
| Yes | 16^2^ (39%) |
| No | 25 (61%) |

^1^Mention of PPI (patient and public involvement), where there was a plan to promote participant retention and PPI was mentioned but it was unclear if PPI members were involved in the designing and planning of the retention strategy specifically that was included as a “possible PPI involvement”. ^2^ 16 protocols mentioned a cost associated with the retention strategy however this was all related to the cost of the monetary incentive/compensation that was going to be used to promote participant retention. Not all monetary incentives mentioned cost.

**List of ‘combined strategies’ outlined in the trial protocols.**

We grouped actions/activities into ORRCA domains based on guidance from ORRCA and from the Cochrane review of strategies to improve retention in clinical trials (2).

Table 3 provides all ‘combined strategies’ (n=128) included in RCT protocols (n=722)

| **Table 3: All combinations of strategies (n=128) outlined in RCT protocols** | | | |
| --- | --- | --- | --- |
|  |  |  |  |
| **Most common combination of retention strategies** | **Number of protocols** | **Most common combo – updated conditional vs unconditional incentives** | **Number of protocols** |
| Non-monetary incentive  Randomisation method  Supporting participation  Reminders   - Monetary incentives | **1** | Non-monetary incentive – Unconditional incentive  Randomisation method  Supporting participation  Reminders   - Monetary incentives - conditional | **1** |
| Monetary incentive   - Reminder | **6** | - Monetary incentive –Conditional incentive - Reminder | **4** |
|  |  | - Monetary incentive – unclear if conditional or not - Reminder | **2** |
| Data collection location and method   - Reminder | **6** |  |  |
| Reminders   - Maintaining engagement | **3** |  |  |
| Prompt  Monetary incentive | **5** | - Prompt - Monetary incentive – conditional incentive | **5** |
| Monetary incentives  Supporting participation | **4** | Monetary incentives – conditional incentive   - Supporting participation | **3** |
|  |  | Monetary incentive – unclear  Supporting participation | **1** |
| Prompts  Supporting participation  Monetary incentive | **1** | Prompts  Supporting participation   - Monetary incentive – conditional incentive | **1** |
| Monetary incentives  Maintaining participant engagement | **1** | Monetary incentives – conditional   - Maintaining participant engagement | **1** |
| Non-monetary incentive  Monetary incentive | **3** | Non-monetary incentive – conditional   - Monetary incentive – conditional | **2** |
|  |  | Non-monetary incentive - unclear  Monetary incentive – conditional | **1** |
| Contact information  Monetary incentives | **2** | - Contact information - Monetary incentives – conditional | **2** |
| Contact information  Maintaining engagement  Monetary incentive | **1** | Contact information  Maintaining engagement   - Monetary incentive – conditional incentive | **1** |
| Supporting participation  Maintaining engagement | **4** |  |  |
| Monetary incentive  Data collection location and method  Reminder | **5** | - Monetary incentive – conditional - Data collection location and method - Reminder | **4** |
|  |  | - Monetary incentive – unconditional - Data collection location and method - Reminder | **1** |
| Supporting participation  Maintaining engagement  Resources and infrastructure | **1** |  |  |
| Prompt  Reminder  Monetary incentive | **4** | - Prompt - Reminder - Monetary incentive – conditional | **4** |
| Prompt  Reminder  Supporting participation | **1** |  |  |
| Prompt  Reminder  Data collection location and method  Monetary incentive | **1** | Prompt  Reminder  Data collection location and method   - Monetary incentive – conditional | **1** |
| Data collection location and method  Contact information  Supporting participation | **1** |  |  |
| Data collection location and method  Maintaining engagement  Reminder | **2** |  |  |
| Prompt  Maintaining participant engagement | **1** |  |  |
| Data collection scheduled with routine care  Data collection location and method | **2** |  |  |
| Supporting participation  Contact information  Prompt  Monetary incentives | **1** | Supporting participation  Contact information  Prompt   - Monetary incentives – conditional |  |
| Trial design  Monetary incentive  Data collection location and method | **1** | Trial design  Monetary incentive – conditional   - Data collection location and method | **1** |
| Data collection location and method  Reminder  Trial design  Maintaining participant engagement | **1** |  |  |
| Prompt  Supporting participation  Data collection location and method  Monetary incentives  Non-monetary incentive | **1** | Prompt  Supporting participation  Data collection location and method  Monetary incentives – unconditional   - Non-monetary incentive – conditional | **1** |
| Monetary incentives  Data collection location and method | **3** | - Monetary incentive – conditional - Data collection location and method | **3** |
| Prompts  Reminder  Data collection with routine care  Data collection location and method | **1** |  |  |
| Cultural consideration  Supporting participation  Reminders | **1** |  |  |
| Data collection location and method  Supporting participation | **1** |  |  |
| Supporting participation  Data collection location and method  Prompt | **1** |  |  |
| Supporting participation  Maintaining participant engagement  Monetary incentives | **1** | Supporting participation  Maintaining participant engagement   - Monetary incentives – conditional | **1** |
| Contact information  Supporting participation  Incentives – conditional but unclear if monetary or non-monetary | **1** |  |  |
| Reminder  Monetary incentive  Non-monetary incentive Data collection location and method | **1** | Reminder  Monetary incentive – conditional  Non-monetary incentive – unconditional   - Data collection location and method | **1** |
| Monetary incentives  Non-monetary incentive  Prompts  Maintaining participant engagement | **1** | Monetary incentives – conditional  Non-monetary incentive – unconditional  Prompts   - Maintaining participant engagement | **1** |
| Maintaining participant engagement  Supporting participation  Non-monetary incentives  Data collection location and method | **1** | Maintaining participant engagement  Supporting participation  Non-monetary incentives – conditional   - Data collection location and method | **1** |
| Acceptability of the protocol  Randomisation method  Questionnaire design  Reminder | **1** |  |  |
| Maintaining participant engagement  Data collection location and method | **1** |  |  |
| Prompt  Supporting participation  Relationship with clinical staff  Contact information | **1** |  |  |
| Reminder  Non-monetary incentive | **2** | - Reminder - Non-monetary incentive – unconditional | **1** |
|  |  | - Reminder - Non-monetary incentive –conditional | **1** |
| Data collection frequency and timing  Contact information  Questionnaire design  Reminder  Prompt  Relationship with clinical staff  Non-monetary incentive  Staff training  Supporting participation | **1** | Data collection frequency and timing  Contact information  Questionnaire design  Reminder  Prompt  Relationship with clinical staff  Non-monetary incentive – unclear if conditional or not  Staff training   - Supporting participation | **1** |
| Prompt  Reminder | **4** |  |  |
| Prompt  Maintaining participant engagement  Non-monetary incentive | **1** | Prompt  Maintaining participant engagement   - Non-monetary incentive – unconditional | **1** |
| Prompt  Reminder  Monetary incentive  Maintaining participant engagement | **1** | Prompt  Reminder  Monetary incentive – conditional   - Maintaining participant engagement | **1** |
| Reminder  Maintaining participant engagement  Supporting participation | **1** |  |  |
| Prompt  Reminder  Data collection location and method | **2** |  |  |
| Maintaining participant engagement  Monetary incentive  Reminder | **1** | Maintaining participant engagement  Monetary incentive – conditional   - Reminder | **1** |
| Maintaining participant engagement  Non-monetary incentive  Monetary incentive  Reminder | **1** | Maintaining participant engagement  Non-monetary incentive – unconditional  Monetary incentive – unconditional   - Reminder | **1** |
| Monetary incentive  Reminder  Supporting participation | **2** | Monetary incentive – conditional  Reminder   - Supporting participation | **2** |
| Reminder  Data collection location and method  Data collection frequency and timing  Supporting participation | **1** |  |  |
| Monetary incentive  Contact information  Prompt  Supporting participation | **1** | Monetary incentive – conditional  Contact information  Prompt   - Supporting participation | **1** |
| Routine data  Maintaining participant engagement  Reminder | **1** |  |  |
| Maintaining participant engagement  Prompt  Supporting participation  Monetary inventive  Non-monetary incentive | **1** | Maintaining participant engagement  Prompt  Supporting participation  Monetary inventive – conditional   - Non-monetary incentive – unconditional | **1** |
| Monetary incentive  Prompt  Reminder  Data collection location and method | **1** | Monetary incentive – conditional  Prompt  Reminder   - Data collection location and method | **1** |
| Maintaining participant engagement  Non-monetary incentive  Monetary incentives | **1** | Maintaining participant engagement  Non-monetary incentive – unconditional   - Monetary incentives – conditional | **1** |
| Maintaining participant engagement  Reminder  Monetary incentive  Non-monetary incentive | **1** | Maintaining participant engagement  Reminder  Monetary incentive – conditional   - Non-monetary incentive – conditional |  |
| Prompt  Maintaining participant engagement  Monetary incentive | **3** | - Prompt - Maintaining participant engagement - Monetary incentive – conditional | **2** |
|  |  | - Prompt - Maintaining engagement - Monetary incentive – conditional - Monetary incentives – unconditional | **1** |
| Prompt – sites and site staff  Prompt - participants  Maintaining participant engagement  Monetary incentive  Data collection location and method | **1** | Prompt – sites and site staff  Prompt - participants  Maintaining participant engagement  Monetary incentive – conditional   - Data collection location and method | **1** |
| Contact information  Monetary incentives  Prompt  Reminder | **1** | Contact information  Monetary incentives – conditional  Prompt   - Reminder | **1** |
| Monitoring approach  Maintaining participant engagement | **1** |  |  |
| Contact information  Prompt  Reminder  Data collection location and method  Non-monetary incentive  Supporting participation | **1** | Contact information  Prompt  Reminder  Data collection location and method  Non-monetary incentive – unconditional   - Supporting participation | **1** |
| Maintaining engagement – site and staff  Monetary incentives  Maintaining participant engagement  Data collection location and method | **1** | Maintaining engagement – site and staff  Monetary incentives – unconditional  Maintaining participant engagement   - Data collection location and method | **1** |
| Monetary incentive  Randomisation method  Acceptability of protocol  Data collection location and method  Supporting participation | **1** | Monetary incentive – conditional  Randomisation method  Acceptability of protocol  Data collection location and method   - Supporting participation | **1** |
| Data collection location and method  Contact information | **1** |  |  |
| Reminder  Supporting participation | **2** |  |  |
| Monetary incentives – conditional  Non-monetary incentives – conditional  Contact information  Reminder | **1** | Monetary incentives – conditional  Non-monetary incentives – conditional  Contact information   - Reminder | **1** |
| Training – sites and site staff category  Contact information | **1** |  |  |
| Choice of study outcomes  Maintaining participant engagement  Reminder | **1** |  |  |
| Prompt  Reminder  Maintaining participant engagement | **1** |  |  |
| Data collection timing and frequency  Reminder  Contact information | **1** |  |  |
| Prompt  Non-monetary incentive | **1** | Prompt   - Non-monetary incentive – unconditional | **1** |
| Prompt  Data collection location and method | **2** |  |  |
| Contact information  Reminder | **1** |  |  |
| Prompt  Maintaining participant engagement  Data collection location and method | **1** |  |  |
| Monetary incentive  Reminder  Contact information  Supporting participation  Data collection frequency and timing | **1** | Monetary incentive – conditional  Reminder  Contact information   - Supporting participation Data collection frequency and timing | **1** |
| Supporting participation  Trial staff  Non-monetary incentive  Trial setting | **1** | Supporting participation  Trial staff  Non-monetary incentive – unconditional   - Trial setting | **1** |
| Reminder  Monetary incentive  Non-monetary incentive | **1** | Reminder  Monetary incentive – conditional   - Non-monetary incentive – unconditional | **1** |
| Monetary incentive  Supporting participation  Data collection location and method | **1** | Monetary incentive – conditional incentive  Supporting participation   - Data collection location and method | **1** |
| Reminder  Data collection location and method  Supporting participation | **1** |  |  |
| Prompt  Supporting participation | **1** |  |  |
| Data collection scheduled with routine care  Maintaining participant engagement | **1** |  |  |
| Trial site factors  Monitoring visits – site and site staff | **1** |  |  |
| Relationship with clinical staff  Maintaining participant engagement | **1** |  |  |

Table 4 provides all ‘combined strategies’ (n=19) included in pilot and feasibility trial protocols

| **Table 4: Combination of retention strategies (n=19) in Pilot and Feasibility trial protocols** | 19 protocols (46.3%) |
| --- | --- |
| **All combinations;** |  |
| Monetary incentives – conditional  Prompt | 2 |
| Monetary incentives – conditional incentive  Supporting participation  Data collection location and method | 2 |
| Data collection location and method  Reminder | 2 |
| Monetary incentives –Conditional incentive  Prompts  Reminders  Acceptability of the protocol | 1 |
| Prompt  Data collection location and method  Supporting participation  Questionnaire design  Non-monetary incentive – conditional | 1 |
| Data collection location and method  Reminder  Maintaining engagement | 1 |
| Monetary incentive – unconditional  Data collection location and method  Reminder | 1 |
| Monetary incentives – conditional incentive  Contact information | 1 |
| Contact information  Supporting participation  Monetary incentives – conditional | 1 |
| Prompt  Maintaining participant engagement | 1 |
| Reminder  Data collection scheduled with routine care | 1 |
| Reminder  Non-monetary incentive – unconditional | 1 |
| Monetary incentive – conditional incentive  Supporting participation | 1 |
| Monetary incentive – conditional  Monetary incentive – unconditional  Acceptability of the protocol | 1 |
| Relationship with clinical staff  Prompt | 1 |
| Prompt  Monetary incentive – conditional  Supporting participation | 1 |

**References**

1. Chan A-W, Tetzlaff JM, Gøtzsche PC, Altman DG, Mann H, Berlin JA, et al. SPIRIT 2013 explanation and elaboration: guidance for protocols of clinical trials. Bmj. 2013;346.

2. Gillies K, Kearney A, Keenan C, Treweek S, Hudson J, Brueton VC, et al. Strategies to improve retention in randomised trials. Cochrane Database of Systematic Reviews. 2021(3).
